# New cobalt hydroxycarbonate-based material for highly sensitive enzyme-free glucose sensors

**DOI:** 10.1038/s41598-025-01164-2

**Published:** 2025-05-17

**Authors:** Zh. K. Kalkozova, L. V. Gritsenko, U. A. Balgimbayeva, M. T. Gabdullin, Dan Wen, Kh. A. Abdullin

**Affiliations:** 1 Institute of Applied Science & Information Technology, Shashkin Str. 40-48, 050040 Almaty, Kazakhstan; 2 National Nanotechnology Laboratory of Open Type of Al-Farabi Kazakh National University, Al-Farabi Ave., 71, 050040 Almaty, Kazakhstan; 3 Satbayev University, Satpaev St., 22, 050013 Almaty, Kazakhstan; 4 Kazakh-British Technical University, Tole Bi Street, 59, 050000 Almaty, Kazakhstan; 5 School of Materials Science and Engineering, Northwestern Polytechnical University (NPU) and Shaanxi Joint Laboratory of Graphene, Xi’an, 710072 People’s Republic of China

**Keywords:** Cobalt hydroxycarbonate, Hydrothermal route, Enzyme-free, Electrochemical sensor, Glucose, Engineering, Materials science, Nanoscience and technology, Physics

## Abstract

Health monitoring with glucose sensors is essential for preventing various diseases and maintaining overall well-being. Among the numerous analytical methods used to measure glucose levels, electrochemical enzyme-free methods show high practical potential. Developing new materials for highly sensitive and stable enzyme-free sensors is of significant importance. This study presents hydroxycarbonates as a new class of materials for creating enzyme-free glucose sensors and demonstrates, for the first time, that nanostructured cobalt hydroxycarbonate is a promising candidate for effective sensor applications. Prepared using a simple one-step hydrothermal method, this material exhibited a sensitivity of 1950 µA cm^−2^ mM^−1^ with a detection limit (LOD) of approximately 30 µM and a linear range up to 3 mM glucose. In addition, the sensitivity of the sensor synthesized in a 0.1 M cobalt nitrate solution was found to increase by more than 3 times up to 6745 µA cm^−2^ mM^−1^ when only 2 mol% of zinc nitrate was added to the growth solution containing cobalt nitrate and urea during synthesis. This adjustment also significantly affects the lattice vibration spectrum and material morphology, reducing the average nanoparticle size from 30 nm to 7–8 nm. The sensors demonstrated high long-term stability when stored under normal conditions. These results highlight the significant potential of the new sensor material for the quantitative detection of glucose.

**Supplementary Information:**

The online version contains supplementary material available at 10.1038/s41598-025-01164-2.

## Introduction

It is well established that glucose levels in the human body are directly linked to cardiovascular disease, weight control and obesity, type 2 diabetes, Alzheimer’s disease, and several other conditions^1,2^. These diseases significantly reduce life expectancy and quality of life, making blood glucose monitoring essential for both clinical diagnosis and personalized health assessment. Glucose monitoring is crucial not only in medical care and clinical analysis but also in food production, quality control of fruit crops, and other fields^1,3^. For practical applications, glucose measurement methods must meet requirements for high accuracy and sensitivity, measurement stability and reproducibility, selectivity, rapid analysis, cost-effective sensor manufacturing, and long shelf life without degradation of the sensor material’s properties.

The most effective methods for measuring glucose concentration in analytes, which have found widespread application, include optical methods (absorption and fluorescence spectroscopy), acoustic methods, and various electrochemical methods^1,4–7^. A comparison of glucose sensors using different analytical methods^5,8,9^ shows that numerous glucose sensing techniques have been developed to analyze glucose levels with the required accuracy. However, these methods vary in the equipment used, analysis procedure, speed of obtaining results, repeatability, selectivity, cost, and other factors that are crucial for practical applications. Acoustic and optical methods typically require more sophisticated equipment and careful sample preparation than other methods, which can increase both the analysis time and cost. Electrochemical methods for measuring glucose levels have found widespread practical application due to their rapidity and low cost^10,11^. These methods are categorized into enzymatic and non-enzymatic techniques^12–17^. Enzymatic sensors operate based on the indirect electrooxidation of glucose molecules, involving oxidoreductase enzymes such as glucose oxidase or glucose dehydrogenase (e.g., hexokinase, glucose oxidase (GOx), or glucose-1-dehydrogenase (GDH))^15,18^. Electrochemical enzymatic glucose measurement methods are well-established in clinical practice and currently dominate the glucose sensor market.

However, enzyme sensors have certain disadvantages: the enzymes used in these sensors degrade over time and can be chemically affected if stored improperly, necessitating regular calibration^15,19^. Non-enzymatic electrochemical methods represent the next generation of sensors^16,19–23^. They eliminate many of the drawbacks associated with enzyme sensors and are currently undergoing intensive development. In non-enzymatic sensors, glucose detection occurs through adsorption on the sensor surface, followed by the electrocatalytic oxidation of glucose at the catalytically active sites of the sensor material^24,25^. Electrochemical enzyme-free glucose measurement methods offer several advantages over other techniques, including high sensor stability, high sensitivity, accuracy, ease of use, quick analysis times, the requirement of only a small analyte volume, and low cost^16,26,27^. This balanced combination of characteristics makes electrochemical enzyme-free glucose measurement methods a potentially preferred choice for both clinical and personal use. Enzyme-free sensors for glucose detection are being developed using a wide range of materials, including noble and transition metals, their alloys and composites, as well as metal compounds, oxides, chalcogenides, and more.

Materials for the fabrication of various enzyme-free glucose sensors are currently being developed intensively, as evidenced by the large number of publications and review papers^24,28–34^. Noble metals and their alloys exhibit high catalytic activity in the non-enzymatic electrooxidation of glucose due to their positive and relatively large reduction potential compared to other metals. Among the noble metals, gold has proven to be an effective material for enzyme-free glucose detectors in alkaline environments, while platinum shows low selectivity and is prone to poisoning by blood components^35^. A wide range of conductive materials, including carbon materials, metals like copper, nickel, cobalt, iron, manganese, bimetallic compounds, oxides, metal sulfides, selenides, conducting polymers, and other carbon materials, are considered promising for electrochemical enzyme-free glucose oxidation and sensor development^3,28,32,33,35–37^. Since the formation of oxidative species is facilitated in an alkaline environment, these sensors typically operate in a slightly alkaline or locally alkaline medium.

The detection mechanism has been detailed in numerous publications^1,5,28^. For instance, with copper, the initial step involves the formation of copper hydroxide on the surface at positive potentials^20^:

1 $${\text{CuO}} + {\text{OH}}^{ - } \to {\text{Cu}}\left( {{\text{OH}}} \right)_{{2}} + {\text{ e}}^{ - }$$

2 $${\text{Cu}}\left( {{\text{OH}}} \right)_{{2}} + {\text{OH}}^{ - } \to {\text{CuOOH}} + {\text{ H}}_{{2}} {\text{O}} + {\text{ e}}^{ - } ,$$

The strong oxidizing agent, CuOOH, then facilitates the oxidation of glucose:

3 $${\text{CuOOH }} + {\text{ C}}_{{6}} {\text{H}}_{{{12}}} {\text{O}}_{{6}} \left( {{\text{glucose}}} \right) \, \to {\text{ Cu}}\left( {{\text{OH}}} \right)_{{2}} + {\text{ C}}_{{6}} {\text{H}}_{{{1}0}} {\text{O}}_{{6}} ({\text{gluconolactone}}) .$$

Similarly, in the case of a cobalt sensor, the stable form of cobalt oxide, Co_3_O_4_, is transformed on the sensor surface into CoO_2_ containing Co(IV) atoms^33,38^

4 $${\text{Co}}_{{3}} {\text{O}}_{{4}} + {\text{OH}}^{ - } + {\text{ H}}_{{2}} {\text{O }} \to {\text{ 3CoOOH }} + {\text{ e}}^{ - } ,$$

5 $${\text{CoOOH }} + {\text{ OH}}^{ - } \to {\text{ CoO}}_{{2}} + {\text{ H}}_{{2}} {\text{O }} + {\text{ e}}^{ - } .$$

These reactions occur in alkaline media at small positive biases at the electrode. The primary contributor to the oxidation of glucose to gluconolactone is the strong oxidizing species CoO_2_, although Co_3_O_4_and CoOOH can also oxidize glucose^38^.

The main characteristics of enzyme-free glucose sensors—such as sensitivity (µA cm^−2^ mM⁻^1^), limit of detection (LOD, µM), sensitivity linear range (mM), working electrolyte, stability, reproducibility, selectivity, etc.—are cited in^1,24,29^ and many other review papers. For example, typical characteristics of Au-based electrochemical non-enzymatic glucose sensors include a linear range of 0.01 to 10–20 mM, an LOD of 1 to 50 µM, and a sensitivity that can reach up to 700 µA cm⁻^2^ mM⁻^1^, with much lower average values^5,28,32^. These parameters allow for the accurate determination of blood glucose levels and serve as benchmarks for most glucose sensors under development. Increasing the sensitivity of sensors and improving the detection limit is a significant challenge, as monitoring glucose levels in biological fluids such as urine, saliva, sweat, tears, and interstitial fluid is crucial for assessing renal function^5^. Diseases that cause high blood glucose concentrations (above 8.8–10 mmol/L) can reduce the kidneys’ reabsorption capacity, resulting in excess glucose being excreted in the urine and potentially appearing in other bodily fluids^17,19^. The concentration of glucose in these fluids is much lower, making high sensor sensitivity vital for accurate disease detection and diagnosis.

Nanoparticles are known to have enhanced catalytic activity compared to bulk materials^20,33^. Therefore, numerous studies focus on developing nanostructured materials for enzyme-free electrochemical glucose sensors to achieve high sensitivity, long-term stability, reduced material consumption, and cost efficiency. The optimal material for enzyme-free electrochemical glucose sensors has not yet been identified, making the development of new materials highly important.

In this work, a material based on cobalt hydroxycarbonate, Co_6_(CO_3_)_2_(OH)_8_^39^—previously used as a battery-type electrode material^40^, for creating electrodes in hybrid supercapacitors^41–43^, and as a material for lithium-ion battery anodes^44^—is proposed for the first time for the development of a glucose sensor. Co_6_(CO_3_)_2_(OH)_8_-based materials have demonstrated catalytic properties for the highly selective electrochemical reduction of carbon dioxide to methanol^45^, as well as for photoelectrochemical oxygen evolution^46^ and the creation of catalysts^47^.

However, to the best of our knowledge, there have been no reports on the use of cobalt hydroxycarbonate as a sensor material until now. In this study, we found that an electrochemical enzyme-free sensor made from the as-synthesized unalloyed material exhibited a sensitivity to glucose of ~ 2000 µA cm⁻^2^mM⁻^1^. Unexpectedly, the synthesis mechanism changed dramatically with the addition of 1% zinc precursor, zinc nitrate (NZn), to the growth solution, resulting in a highly dispersed material with enhanced catalytic activity. Simultaneously, a significant change in the morphology of the samples and their electrochemical properties was observed, leading to a threefold increase in the sensitivity of the sensor made from this material. The new material for glucose sensors demonstrated high stability, with no degradation of its sensitive properties after aging for three months under normal storage conditions. This suggests its potential as a promising sensor material for the determination of glucose concentration within the range of 0–3 mM, with a sensitivity of more than 6 mA cm⁻^2^ mM⁻^1^ and a limit of detection of around 16 µM.

## Material and methods

Zinc nitrate hexahydrate Zn(NO_3_)_2_·6H_2_O (Sigma Aldrich, St. Louis, Missouri, USA), cobalt (II) nitrate hexahydrate Co(NO_3_)_2_·6H_2_O (Sigma Aldrich, St. Louis, Missouri, USA), urea CH_4_N_2_O (Sigma Aldrich, St. Louis, Missouri, USA), nickel foam, acetone, ethanol. Distilled water 18.2 MOhm × cm (ARIUM 611 DI water purification system, Sartorius Group).

Co_6_(CO_3_)_2_(OH)_8_·H_2_O nanocrystals were grown by a simple hydrothermal method. Urea CH_4_N_2_O, cobalt nitrate Co(NO_3_)_2_·6H_2_O (NCo) and zinc nitrate Zn(NO_3_)_2_·6H_2_O (NZn) were used to prepare the solution for growing the samples. The molar concentrations of cobalt nitrate and urea in all syntheses were 0.1 M and 0.4 M, respectively. Sample #1 was synthesized without the addition of zinc nitrate. In contrast, for the other synthesized samples, the ratios of molar concentrations of cobalt nitrate to zinc nitrate in the growth solution varied as shown in Table 1.

**Table 1:** Molar concentrations of growth solution used in synthesis.

Sample	Molar concentration of urea in growth solution	Molar concentration of cobalt in growth solution	Molar concentration of zinc in growth solution	Ratio of zinc and cobalt concentrations
#1	0.4	0.1	0.000	0.00
#2	0.4	0.1	0.001	0.01
#3	0.4	0.1	0.002	0.02
#4	0.4	0.1	0.005	0.05
#5	0.4	0.1	0.009	0.09
#6	0.4	0.1	0.014	0.14
#7	0.4	0.1	0.020	0.20
#8	0.4	0.1	0.033	0.33
#9	0.4	0.1	0.050	0.50
#10	0.4	0.1	0.067	0.67
#11	0.4	0.1	0.091	0.91

Hydrothermal synthesis was performed in a steel autoclave with an inner fluoroplastic liner. The autoclave was hermetically sealed and placed in a preheated muffle furnace set to a synthesis temperature of 140 °C. The synthesis lasted for 4 hours, after which the autoclave was allowed to cool to room temperature. The synthesized samples were then washed several times with deionized water and dried in air at 80 °C in a desiccator. The chemical reactions occurring during the hydrothermal synthesis of Co_6_(CO_3_)_2_(OH)_8_ can be described by the following equations. ^41^:

6 $${\text{CO}}\left( {{\text{NH}}_{{2}} } \right)_{{2}} + {\text{ H}}_{{2}} {\text{O }} \to {\text{ 2NH}}_{{3}} + {\text{ CO}}_{{2}} \uparrow$$

7 $${\text{NH}}_{{3}} + {\text{ H}}_{{2}} {\text{O }} \to {\text{ NH}}^{{{4} + }} + {\text{ OH}}^{ - }$$

8 $${\text{CO}}_{{2}} + {\text{ H}}_{{2}} {\text{O }} \to {\text{ 2H}}^{+} \, + {\text{ CO}}_{{3}}^{{{2} - }}$$

9 $${\text{6Co}}^{{{2} + }} + {\text{ 8OH}}^{ - } + {\text{ 2CO}}_{{3}}^{{{2} - }} + {\text{ H}}_{{2}} {\text{O }} \to {\text{ Co}}_{{6}} \left( {{\text{CO}}_{{3}} } \right)_{{2}} \left( {{\text{OH}}} \right)_{{8}} \cdot{\text{H}}_{{2}} {\text{O}}.$$

The surface morphology and microstructure of the samples were examined using a Quanta 3D 200i FEI scanning electron microscope (SEM). X-ray diffraction (XRD) analysis was performed with a MiniFlex Rigaku diffractometer. Raman spectra were recorded using an Ntegra Spectra (NT-MDT) spectrometer with excitation at a wavelength of 473 nm.

Electrochemical measurements, including cyclic voltammetry (CV) and electrochemical impedance spectroscopy (EIS), were conducted using an Elins P-40X potentiostat with an FRA-24M electrochemical impedance measurement module. These measurements were carried out in a conventional three-electrode electrochemical cell, with a platinum electrode as the counter electrode, an Ag/AgCl electrode as the reference electrode, and a glass-graphite rod (3 mm in diameter) as the working electrode, onto which the investigated powder was applied. The electrochemical measurements were performed in a 0.1 M KOH solution at room temperature.

## Results and discussion

**Fig. 1:**
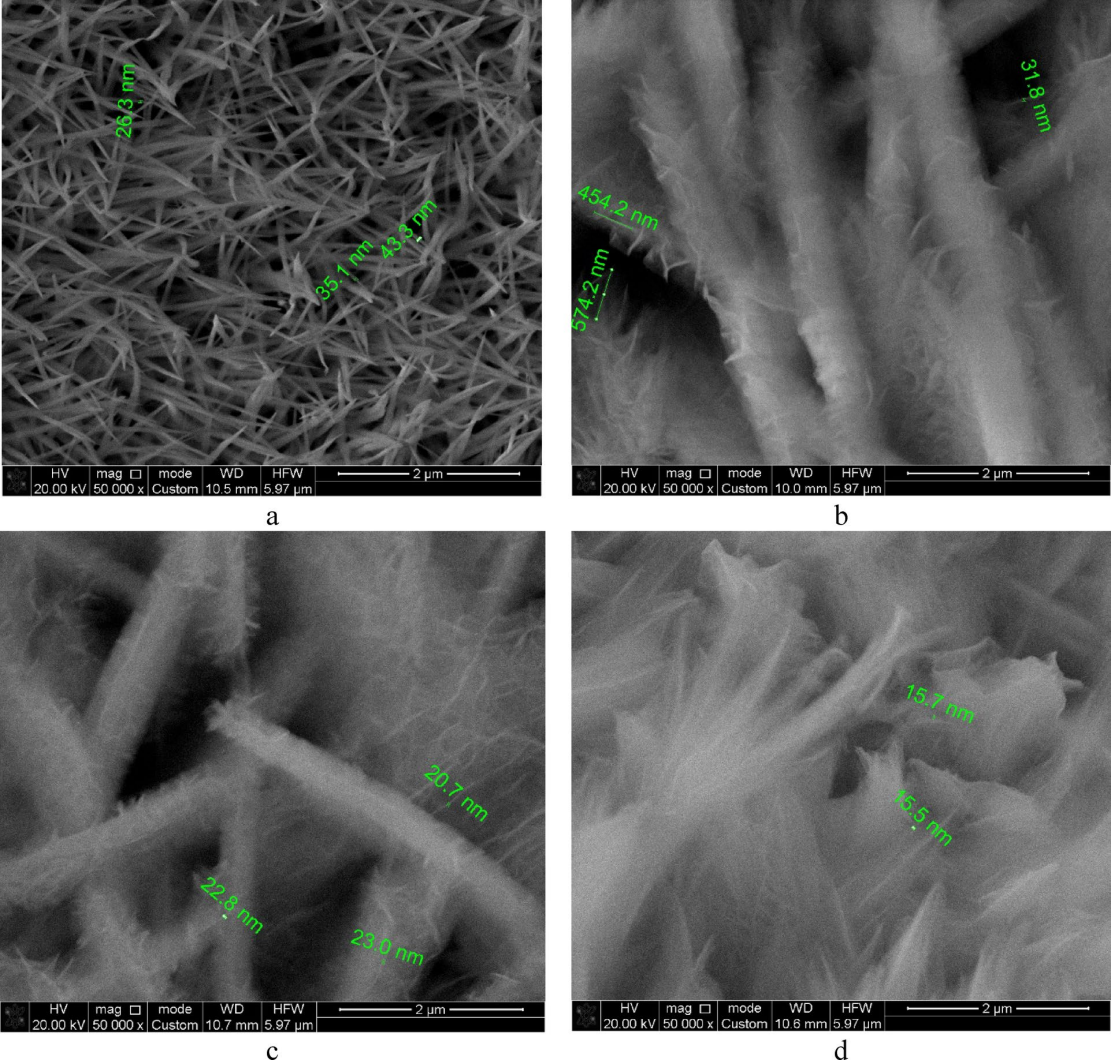
SEM as a function of growth solution composition: (**a**) sample #1, (**b**) – sample #2, (**c**) sample #3, (**d**) sample #4.

The morphology of all synthesized samples was examined using scanning electron microscopy (SEM). Samples grown from a cobalt nitrate-based solution exhibited long, thin rods approximately 35 nm in thickness (sample #1, Fig. 1a). This morphology is characteristic of cobalt hydroxycarbonate and has been observed in previous studies^41,43,44^. It was found that even minor additions of NZn to the growth solution resulted in significant changes in the samples’ morphology. For samples #2 and #3, with NZn concentrations of 1% and 2%, respectively, a much more highly dispersed structure consisting of multiple whiskers approximately 20 nm thick was formed (sample #2, Fig. 1b; sample #3, Fig. 1c). Increasing the NZn concentration further led to the formation of fibers approximately 15 nm thick, which were packed into bundles (sample #4, Fig. 1d), and the creation of extended plates approximately 30–40 nm thick (Supplementary Fig. S1).

**Fig. 2:**
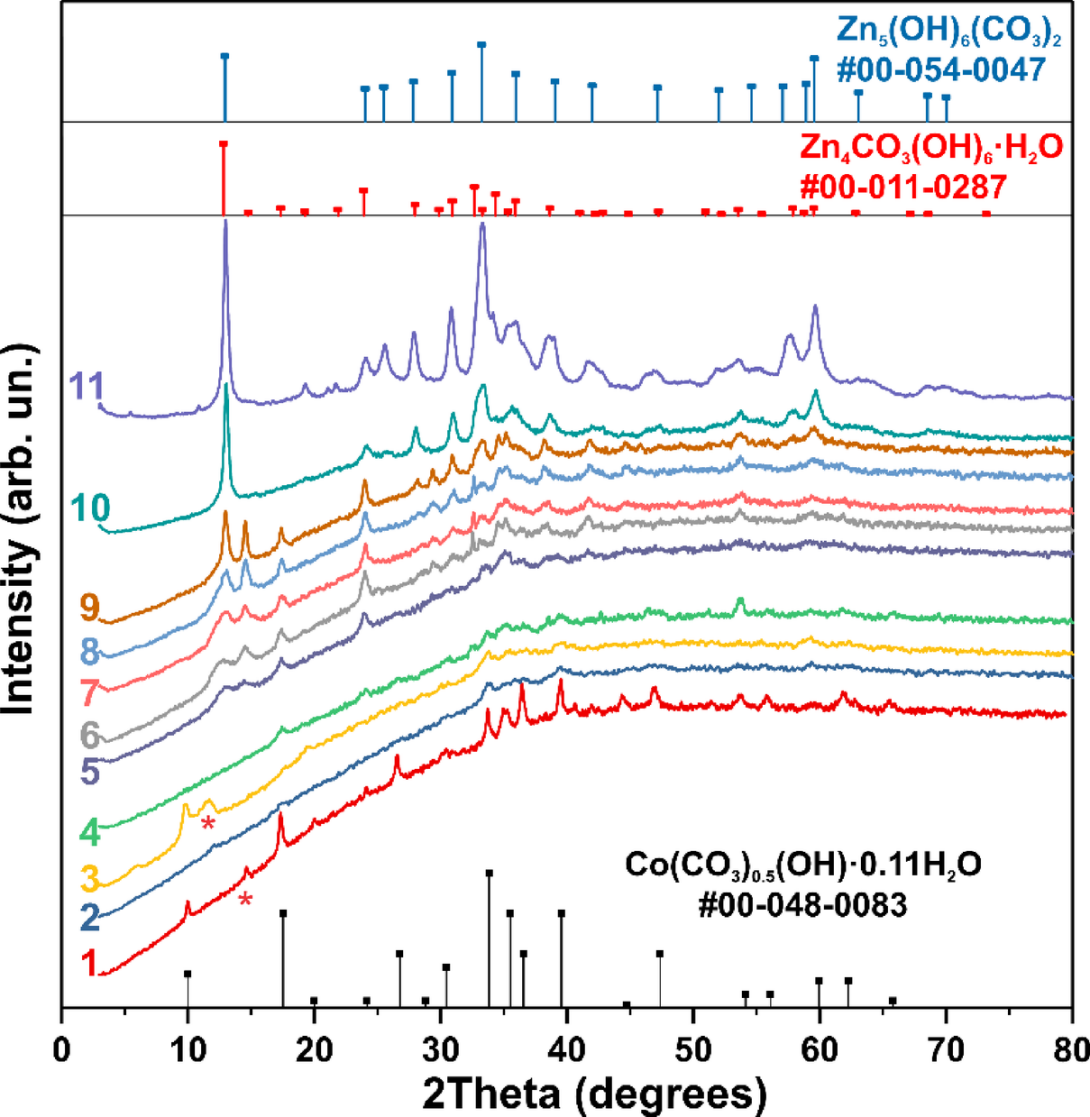
XRD analysis as a function of growth solution composition.

Energy dispersive X-ray spectroscopy (EDX) was used to determine the elemental composition of the samples and to map their distribution (Supplementary Fig. S2–S4). The elemental analysis confirmed an increase in zinc concentration in the synthesized samples as the molar content of zinc in the growth solution was raised. Furthermore, elemental mapping images revealed that carbon, oxygen, and zinc atoms are uniformly distributed on the surface of the samples, while the distributions of cobalt and zinc do not overlap (Supplementary Fig. S2–S4), indicating that no joint phase is formed. This observation, along with the significant increase in the dispersibility of the synthesized material upon the addition of NZn to the growth solution, is supported by the XRD results. XRD data for sample #1 indicates that it consists of a cobalt hydroxycarbonate phase, with all observed reflections matching PDF Card No.: 00-048-0083, previously assigned to the structure Co(CO_3_)_0.5_(OH)·0.11H_2_O. However, a subsequent study^39^ based on synchrotron powder diffraction data revised the chemical formula to Co_6_(CO_3_)_2_(OH)_8_·H_2_O. The addition of even a small concentration of NZn to the growth solution results in a sudden change in the XRD patterns, similar to the abrupt change in morphology. For samples #2, #3, and #4, synthesized with 1%, 2%, and 5% NZn in the growth solution, respectively, the cobalt hydroxycarbonate peaks are extremely weak and broadened, indicating that the synthesis of the cobalt compound is disrupted by the presence of NZn. No compounds incorporating both cobalt and zinc are formed. This suggests a significant change in the growth mechanism and suppression of the synthesis of the Co₆(CO₃)₂(OH)₈·H₂O structure. At a zinc precursor concentration of approximately 9% in the solution, the formation of zinc hydroxycarbonates is observed (Fig. 2, curve 5), including phases Zn₄CO₃(OH)₆·H₂O (PDF Card No.: 00–011-0287) and Zn₅(OH)₆(CO₃)₂ (PDF Card No.: 00-054-0047).

**Fig. 3:**
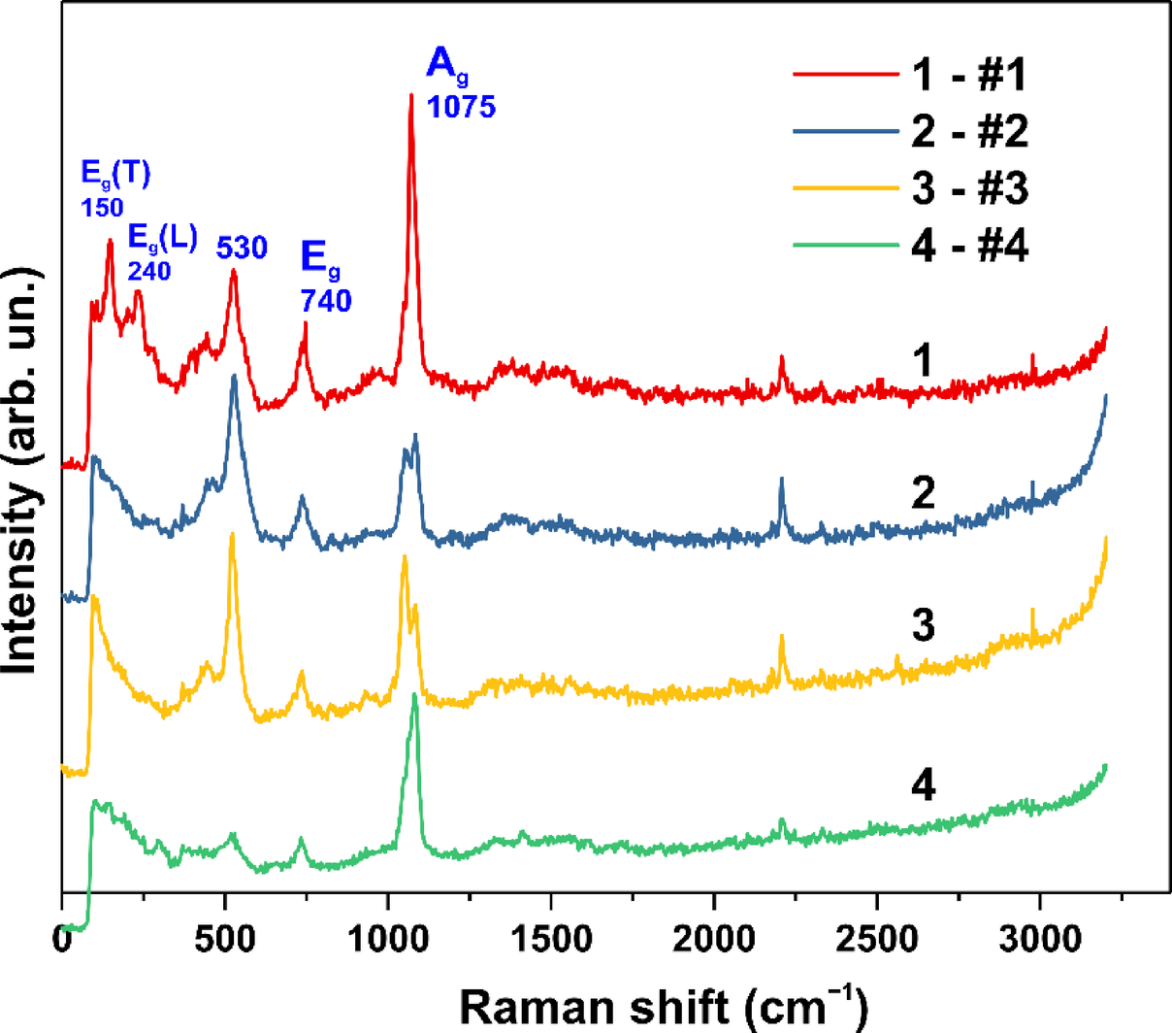
Raman spectra of samples #1, #2, #3, and #4 (curves 1–4, respectively).

The structural changes in Co_6_(CO_3_)_2_(OH)_8_·H_2_O during synthesis at low concentrations of NZn were also observed in the Raman spectra. The Raman spectral data for samples #1, #2, #3, and #4 are shown in Fig. 3. An intense peak at ~ 1075 cm⁻^1^ is present in all the spectra. This peak in sample #1 indicates the presence of Co–(CO₃) groups, a feature commonly observed in metal carbonates^48^. This peak corresponds to the symmetric stretching mode, with its frequency varying between ~ 1070–1095 cm⁻^1^ due to metal substitution and changes in the bond strength force constant^49^. The peak at ~ 740 cm⁻^1^ can also be attributed to the in-plane bending vibrations of the Co-(CO₃) group, while the peaks at 150 and 240 cm⁻^1^ are associated with lattice vibrations^48^, and the peak at ~ 530 cm⁻^1^ may correspond to Co(OH)₂ group vibrations^50^. Notably, the low-frequency lattice modes visible in sample #1, cobalt hydroxycarbonate Co_6_(CO_3_)_2_(OH)_8_·H_2_O, disappear when synthesis is conducted in the presence of NZn (Fig. 3, curve 2). This disappearance correlates with the loss of cobalt hydroxycarbonate phase reflections in XRD data (Fig. 2) and indicates a significant change in the crystal structure. Raman band splitting of the Ag mode upon doping (Fig. 3, spectra 2 and 3) reveals the presence of two types of Me-(CO₃) groups, corresponding to vibrations of Zn-(CO₃) and Co–(CO₃) at 1055 cm⁻^1^ and 1085 cm⁻^1^, respectively. Thus, SEM, XRD, and Raman data collectively show that even minor additions of zinc precursors to the growth solution dramatically alter the crystal structure and morphology of the synthesized samples. Similar dramatic changes were observed in the sensory properties of these materials.

**Fig. 4:**
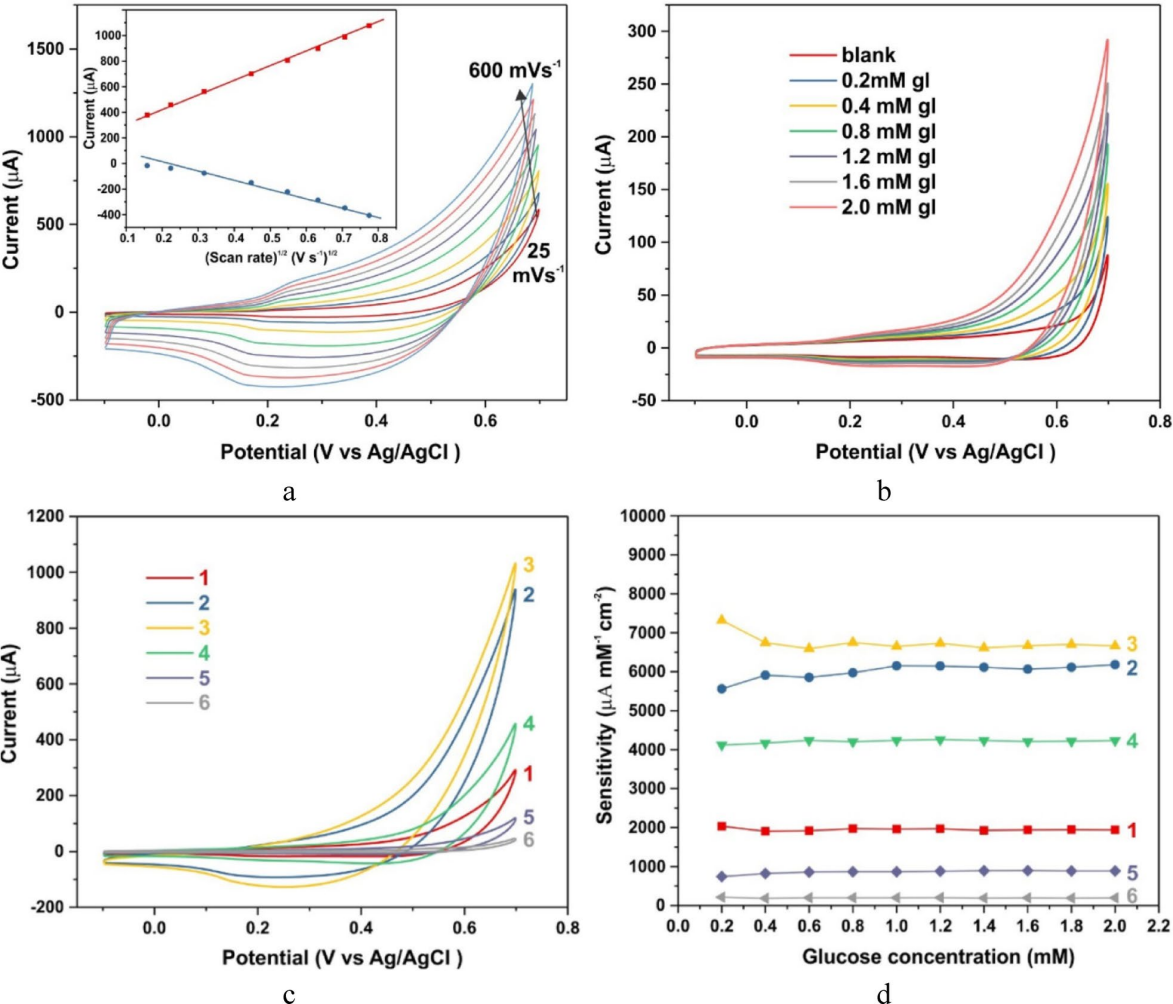
Cyclic voltammetry. (**a**) CV curves for sample #3 Zn-0.02 at a glucose concentration of 2 mM and various potential sweep rates (25, 50, 100, 200, 300, 400, 500, and 600 mV·s⁻^1^). The inset shows the current vs. square root of scan rate for both the forward and reverse branches at a potential of 0.6 V. (**b**) CV curves for sample #1 Zn-0 (cobalt only) at a potential sweep rate of 100 mV s⁻^1^, plotted as a function of glucose concentration. (**c**) CV curves for samples #1 Zn-0 (curve 1), #2 Zn-0.01 (curve 2), #3 Zn-0.02 (curve 3), #4 Zn-0.05 (curve 4), #8 Zn-0.33 (curve 5), and a glassy carbon electrode (GCE) (curve 6) at a glucose concentration of 2 mM and a scan rate of 100 mV s⁻^1^. (**d**) Sensitivity of the samples shown in panel c as a function of glucose concentration.

To determine the sensitivity of the synthesised samples towards glucose, their electrochemical properties were investigated in a three-electrode electrolytic cell in an alkaline solution of 0.1 M KOH at a potential of 0.6 V versus Ag/AgCl. A glass-graphite electrode was used as the working electrode. With stepwise addition of glucose, the anodic CV scanning current increased, suggesting enhanced oxidation of glucose at the electrode. Figure 4 a shows the effect of CV scan rate between 25 and 600 mV s^−1^ on the value of the maximum oxidation current of sample #3 at 2 mM glucose concentration. The inset to Fig. 4 a shows the dependence of the anodic and cathodic currents on the root of the scan rate. The observed linear relationship between the current and the square root of the scan rate suggests that the electrochemical redox reaction proceeded predominantly due to the diffusion of glucose on the surface of the working electrode. Figure 4b shows the cyclic voltammetry (CV) curves of sample #1 at different glucose concentrations in alkaline solution. Similar mean CV curves were obtained for all samples of the considered series (Supplementary Figure S5). The sensitivity of all samples was calculated based on the dependence of oxidation current on glucose concentration (Supplementary Figure S6) as the tangent of the slope of the curve. The highest sensitivity was exhibited by samples #2 and #3 grown at concentrations of 1% and 2% NZn (Fig. 4c,d), the sensitivity reaches a maximum value of 7000 µA cm^−2^ mM^−1^ for samples synthesised at 2% NZn concentration and thereafter the sensitivity decreases rapidly.

**Fig. 5:**
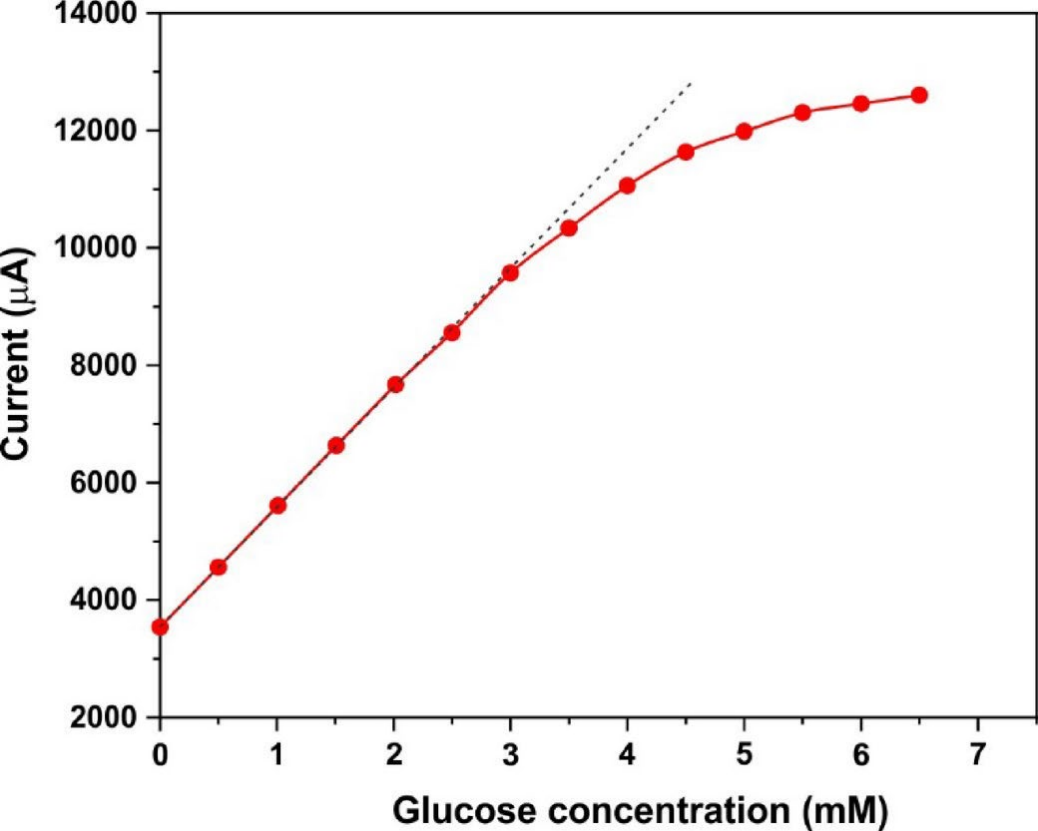
The dependence of current vs. the glucose concentration for sample #3.

The linear relationship between sensitivity and glucose concentration is maintained up to 3 mM glucose (Fig. 5), which meets the requirements for detecting glucose in human serum samples under physiological conditions^51^. Even at higher concentrations, the sensitivity remains sufficiently high. The limit of detection (LOD) was calculated using the formula 3σ/slope, where σ is the standard deviation of the current density and the slope is from the calibration curve^52^. The LOD for the synthesized samples ranged from 16.1 to 38.2 µM.

Amperometric measurements with the glassy carbon electrode (GCE) using sample #3 for glucose detection at an applied potential of 0.6 V demonstrated a rapid and linear response (Supplementary Figure S7). Electrochemical impedance spectroscopy (EIS) was performed in the frequency range of 0.1–10^5^ Hz to investigate charge transfer efficiency and charge separation at the interface.

**Fig. 6:**
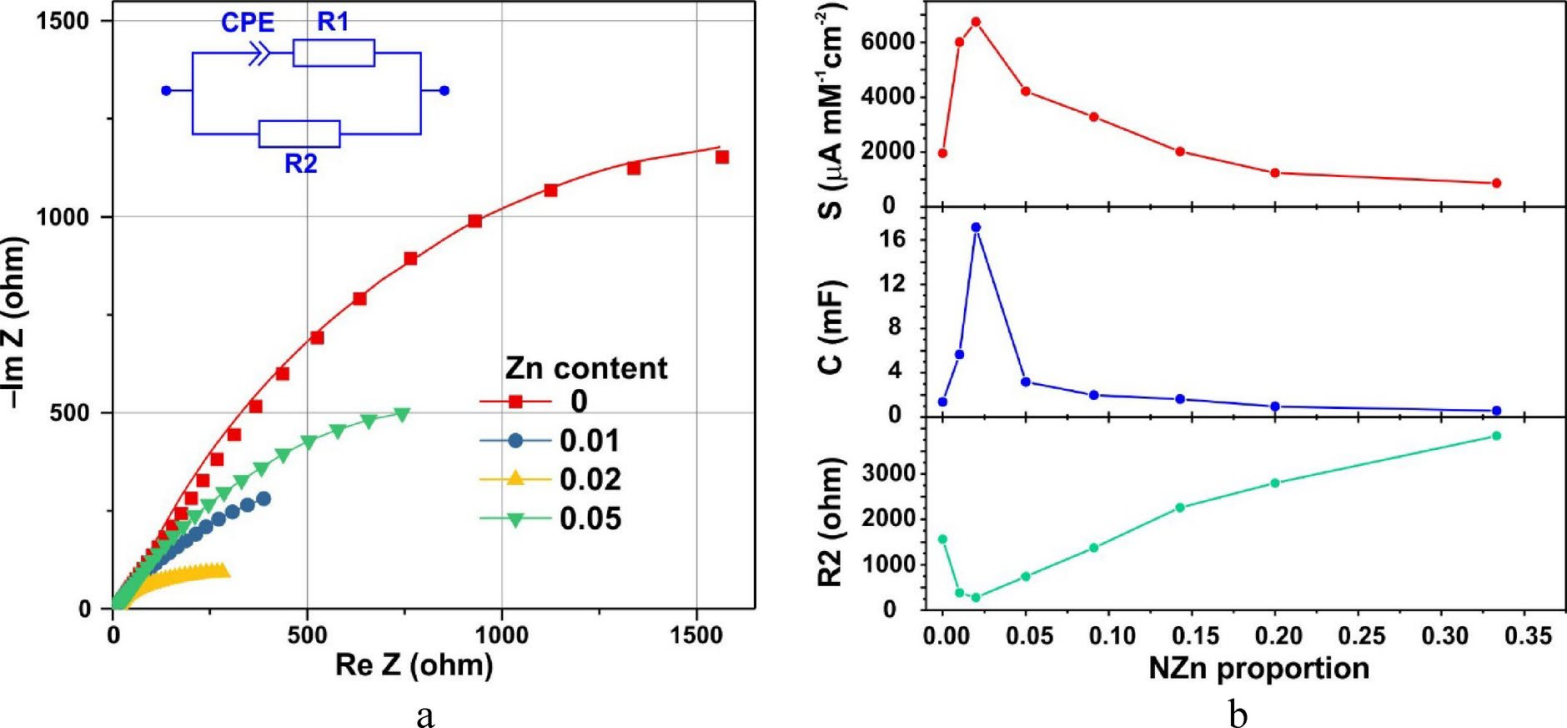
(**a**) Nyquist plots for samples synthesized with molar contents of NZn in the growth solution ranging from 0 to 0.05 relative to the molar content of NCo. The solid lines represent calculations based on the equivalent circuit with CPE, shown in the inset of Fig. 5a; (**b**) the sensitivity S, low-frequency capacitance C of the constant phase element, and resistance R2 as a function of the proportion of NZn in a growth solution containing both NCo and NZn.

The Nyquist plots for the obtained samples (Fig. 6a) exhibit curves close to circles centered at positive values of Im Z, indicating that the equivalent circuit of the electrode can be approximated by a constant phase element (CPE). Accordingly, the fitting of these Nyquist plots was conducted using the equivalent circuit shown in the inset of Fig. 6a. The CPE represents a frequency-dependent complex impedance^53^ that can be approximated by the formula:

10 $${Z}_{CPE}=\frac{1}{{(i\omega )}^{\alpha }Q}=\frac{1}{{(\omega )}^{\alpha }Q}{e}^{-(\frac{\pi }{2}\alpha i)},$$

where α can vary from 0 to 1. At f = 0.16 Hz, the angular frequency ω = 2πf = 1, so the value of Z_*CPE*_ is independent of α at this frequency, and the value of Q at low frequencies can characterize the equivalent capacitance of the CPE element. A comparison of the sensitivity and capacitance of the synthesized samples as a function of NZn content in the growth solution is shown in Fig. 6b. It is evident that there is a direct correlation between sensor sensitivity and CPE capacitance, as both parameters are largely influenced by the specific surface area of the sample. The value of R1, corresponding to the series resistance of the electrolyte and electrode, was approximately the same for all samples, ranging from 8 to 12 ohms. In contrast, R2, which represents the resistance related to the reaction, varied significantly depending on the NZn content in the growth solution and correlated well with changes in sensitivity (Fig. 6b).

**Fig. 7:**
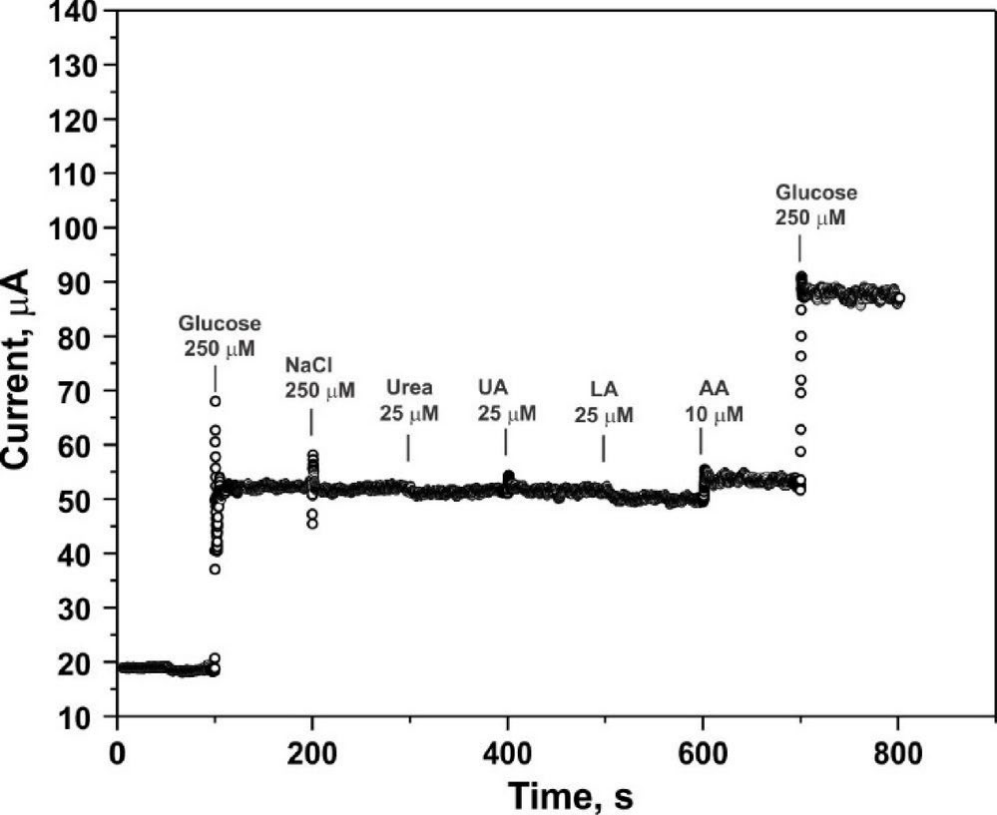
Amperometric response of the sensor (sample #3) to the sequential addition of glucose (0.25 mM), NaCl (0.25 mM), urea (0.025 mM), uric acid (0.025 mM), lactic acid (0.025 mM), ascorbic acid (0.01 mM), followed by a second glucose injection (0.25 mM).

For glucose-level sensors, it is essential to evaluate the effect of interfering substances that can be oxidized at positive potentials, potentially generating false signals and thereby reducing the sensor’s sensitivity. In this study, NaCl, urea, uric acid (UA), lactic acid (LA), and ascorbic acid (AA) were used as representative interfering species. Figure 7 displays the amperometric responses of the sensor based on sample #3, measured at a potential of 0.6 V vs Ag/AgCl, upon the introduction of each compound. The minimal current changes observed in response to these interferents, compared to the signal generated by glucose, demonstrate the good selectivity of the glucose sensor based on the newly developed material.

The glucose sensitivity of Co_6_(CO_3_)_2_(OH)_8_·H_2_O—based material, first discovered in this work, is attributed to the formation of highly active cobalt species during electrochemical oxidation. This is evidenced by the oxidation peak observed at a potential of 0.2–0.3 V (Fig. 4a), corresponding to the Co^2+^ → Co^3+^ transition. The efficiency of the glucose oxidation reaction is enhanced by the high dispersibility of the synthesized material (see SEM figure), and the sensitivity of the sensor increases significantly with the addition of NZn to the growth solution. This effect warrants further discussion. The sharp change in all characteristics upon introducing NZn into the growth solution is notable. A clear correlation is observed between the dramatic changes in XRD patterns and their electrochemical properties, such as sensor sensitivity S, capacitance C of the constant phase element, and reaction resistance R2 (Fig. 6b).

The electrochemical characteristics of the material undergo significant changes that correspond with a sharp alteration in the crystal structure of cobalt hydroxycarbonate. In the unalloyed sample, the crystallite sizes estimated from the half-width of XRD reflections were 29 ± 5 nm. However, with the introduction of a small amount of NZn into the growth solution, the half-width of the XRD reflections for the Co_6_(CO_3_)_2_(OH)_8_·H_2_O phase increases significantly. The estimated crystallite sizes, derived from the half-width of XRD reflections at 10–12° angles (Fig. 2, curve 3), were 8 and 7 nm. The weaker reflections observed in the figure (Fig. 2, curves 2 and 4) correspond to nanoparticles of the same or even smaller size. Despite the slower growth of the crystal structure, no new significant phases appear (Fig. 2), and the synthesis rate and material yield remain unaffected by doping. The noticeable disappearance of lattice lines in the Raman spectra of samples with 1–5% NZn addition (Fig. 3, spectra 2–4) further indicates an increase in the material’s dispersibility. It can be concluded that introducing small amounts of NZn during the synthesis process is an effective way to form Co_6_(CO_3_)_2_(OH)_8_·H_2_O nanocrystals. This addition disrupts the crystal growth of the Co_6_(CO_3_)_2_(OH)_8_·H_2_O phase, resulting in the formation of highly dispersed nanocrystalline and amorphous phases. This conclusion is supported by SEM and Raman results, as well as by the low-intensity X-ray diffraction and broad XRD reflections observed. The nanocrystalline and amorphous states can enhance the material’s catalytic activity compared to its crystalline state. This improvement is primarily due to the increased specific surface area of the material, which consists of nanoparticles with hollow or small-sized morphologies. Additionally, the catalytic activity may increase because of the high degree of unsaturated coordination and the presence of broken bonds, leading to a greater number of active centers. Numerous examples of enhanced electrocatalytic properties in amorphous forms, particularly hydroxides such as cobalt hydroxides, are discussed in the review by Xiuyi Yang et al.^54^. Table 2 presents a comparison of the sensor characteristics of the developed cobalt hydroxycarbonate-based glucose electrode with other non-enzymatic electrodes. The proposed Co₆(CO₃)₂(OH)₈·H₂O sensor exhibits high sensitivity for glucose detection.

**Table 2:** Comparison of the developed cobalt hydroxycarbonate glucose electrodes with other non-enzymatic glucose sensor electrodes.

Glucose sensor’s materials	Linear range	Sensitivity (µA × mM^−1^ × cm^−2^)	Detection limit (μM)	References
Nickel-LIG	12 μM-1.5 mM	5796.18	0.0152	^9^
ZnO NW/ITO	3 mM	92	–	^23^
CuO/Ag/NiO	0.001–5.50 mM	2895.3	0.1	^36^
Cu@Ni	0.2–12.2 mM	420	62.5	^37^
Co-MOF/GCE	0.005–0.9 mM	0.169	1.6	^55^
GO/Co(OH)_2_	0.1 μM-8.546 mM	2412.7	0.934 M	^56^
CuO MF-SPE	6 mM	383	10	^57^
CoNPs/ITO electrode	0.005–0.18 mM	1720	0.25	^58^
Co_3_O_4_ nanoparticles	up to 0.5 mM	33,245	5	^59^
ZnO/Co_3_O_4_/rGO	0.015–10 mM	1551.38	0.043	^60^
CONM/GC	0.7–60 µM	2515.35	0.15	^61^
aMC-CoPc/PI	0.1–3.5 mM	22.3	27.4	^62^
Co_3_O_4_/SWCNT	1–5 mM	96.92	0.25	^63^
A hollow Mn–Cu–Al oxide nanocomposite	5 μM–2.5 mM	2194	0.43	^64^
Au/Co_3_O_4_/FTO	0.2 μM–20 mM	6000	0.1	^65^
NHCN–Co_3_O_4_	1.0 μM–32.0 mM	–	0.2	^66^
NiO	0.1–1 mM	1202	0.25	^67^
Co_6_(CO_3_)_2_(OH)_8_·H_2_O	3 mM	6745	16	This work

63

**Fig. 8:**
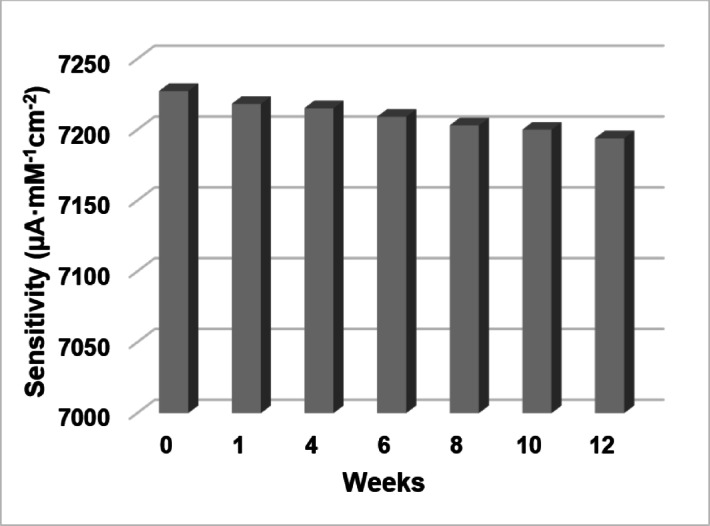
Aging of sample #3.

All synthesized samples demonstrated excellent stability in their sensitivity towards glucose. Figure 8 illustrates the variation in the average sensitivity of sample #3 over a 12-week period. During three months of storage under normal conditions, the sensitivity of all synthesized samples decreased by only 0.5% on average.

Thus, the formation of highly dispersed material explains the non-monotonic dependence of the electrocatalytic properties of the synthesized materials. At the same time, this synthesis method is effective in achieving higher catalytic activity.

## Conclusions

In this paper, we demonstrate for the first time that hydroxycarbonates represent a new class of materials suitable for creating electrochemical sensors. Using cobalt hydroxycarbonate, Co_6_(CO_3_)_2_(OH)_8_·H_2_O, synthesized through a simple hydrothermal method, we present an effective enzyme-free electrochemical glucose sensor. The sensor exhibits a high sensitivity of 1950 µA cm^−2^ mM^−1^ at a potential of 0.6 V vs. Ag/AgCl, with a linear range up to 3 mM glucose and a detection limit of 30 µM, demonstrating excellent stability during storage under ambient conditions. Another important discovery is that even a small concentration of zinc precursors in the growth solution disrupts the growth mechanism of the Co_6_(CO_3_)_2_(OH)_8_·H_2_O phase, leading to the formation of a highly dispersed nanocrystalline phase with a high specific surface area. As a result, there is a significant improvement in catalytic activity and an increase in sensor sensitivity to 6745 µA cm^−2^ mM^−1^, with a detection limit of 16 µM, achieved by increasing material dispersion and reducing nanoparticle size. The synthesized material demonstrated excellent stability in its sensory properties, with only a 0.5% change in sensitivity over 12 weeks of storage under ambient conditions. These results highlight the potential of this new sensor material, synthesized through a simple and efficient one-step hydrothermal method, for accurate glucose detection.

## Electronic supplementary material

Below is the link to the electronic supplementary material.

Supplementary Material 1

## Data Availability

All data generated or analysed during this study are included in this published article.
