# Supplementary material for: New cobalt hydroxycarbonate-based material for highly sensitive enzyme-free glucose sensors
Source: Sci Rep. 2025 May 17;15:17154. doi: 10.1038/s41598-025-01164-2 (PMC12085578; doi:10.1038/s41598-025-01164-2)
Supplement: Supplementary file 1 — Supplementary Material 1 [file 41598_2025_1164_MOESM1_ESM.pdf]

scientific reports

Supplementary

OPEN

New Cobalt Hydroxycarbonate-Based Material for Highly Sensitive Enzyme-Free Glucose Sensors

Zh.K. Kalkozova[1,2], L.V. Gritsenko[1,3]✉, U.A. Balgimbayeva[4], M.T. Gabdullin[4], Dan Wen[5], Kh.A. Abdullin[1,2]✉

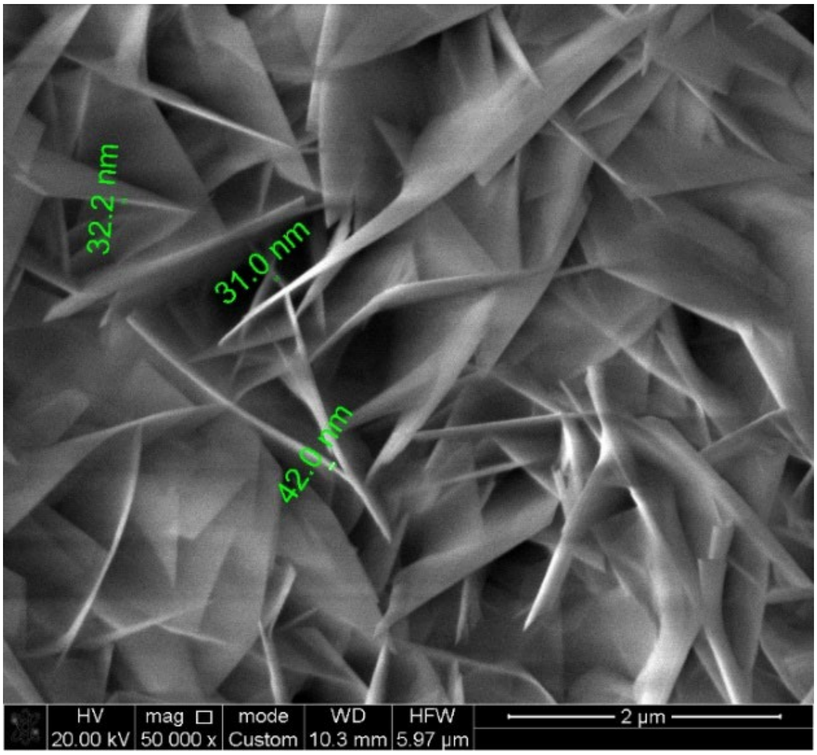

Fig. S1. SEM image for sample #2.

[1]Institute of Applied Science & Information Technology, Shashkin Str. 40-48, Almaty 050040, Kazakhstan. [2]National Nanotechnology Laboratory of Open Type of Al-Farabi Kazakh National University, al-Farabi ave., 71, Almaty 050040, Kazakhstan. [3]Satbayev University, Satpaev St., 22, Almaty 050013, Kazakhstan. [4]Kazakh-British Technical University, Tole bi Street, 59, Almaty 050000, Kazakhstan. [5] School of Materials Science and Engineering, Northwestern Polytechnical University (NPU) and Shaanxi Joint Laboratory of Graphene, Xi'an 710072, P. R. China. ✉email: l.gritsenko@satbayev.university, kh.abdullin@physics.kz (K.A.A.)

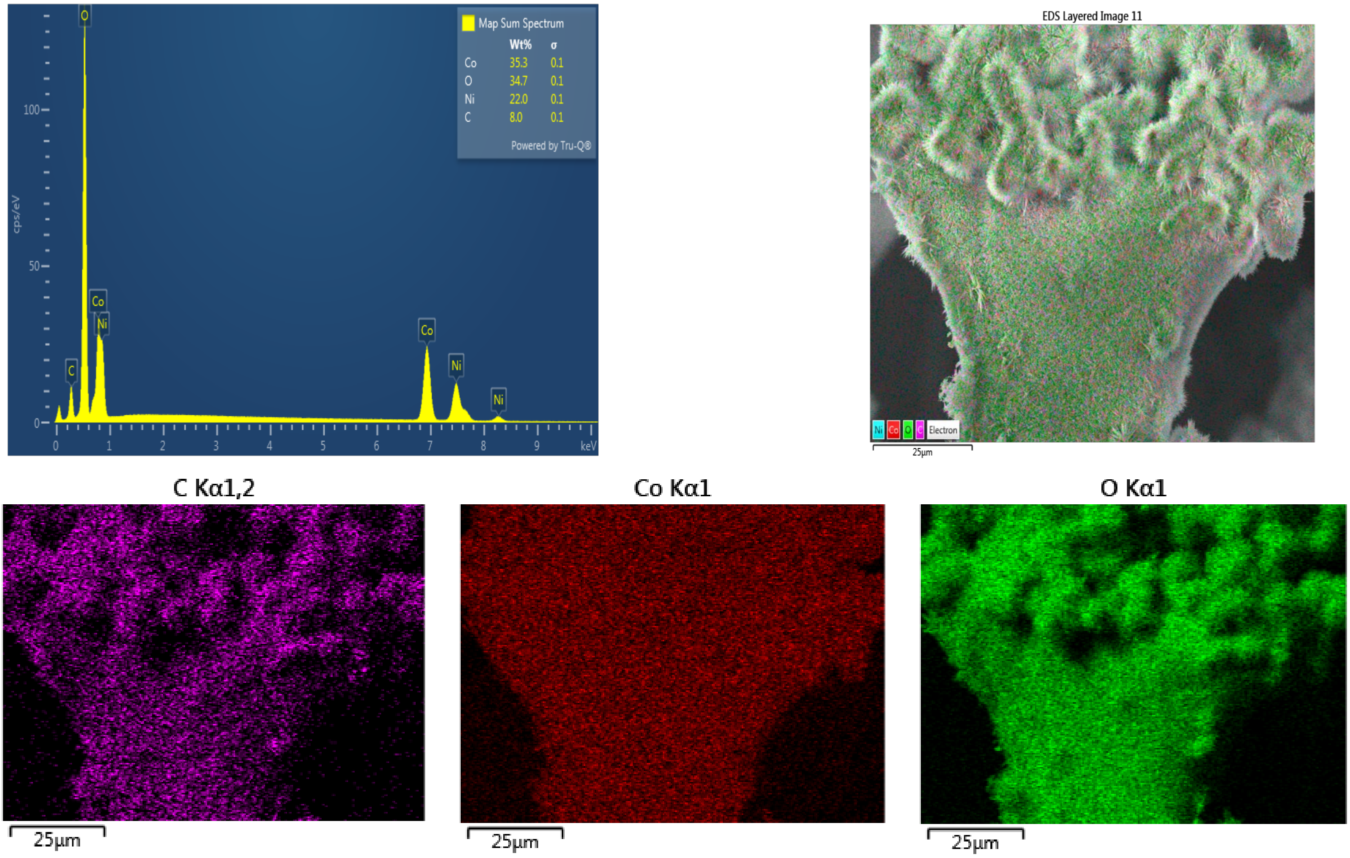

Fig. S2. EDS graph for sample #1.

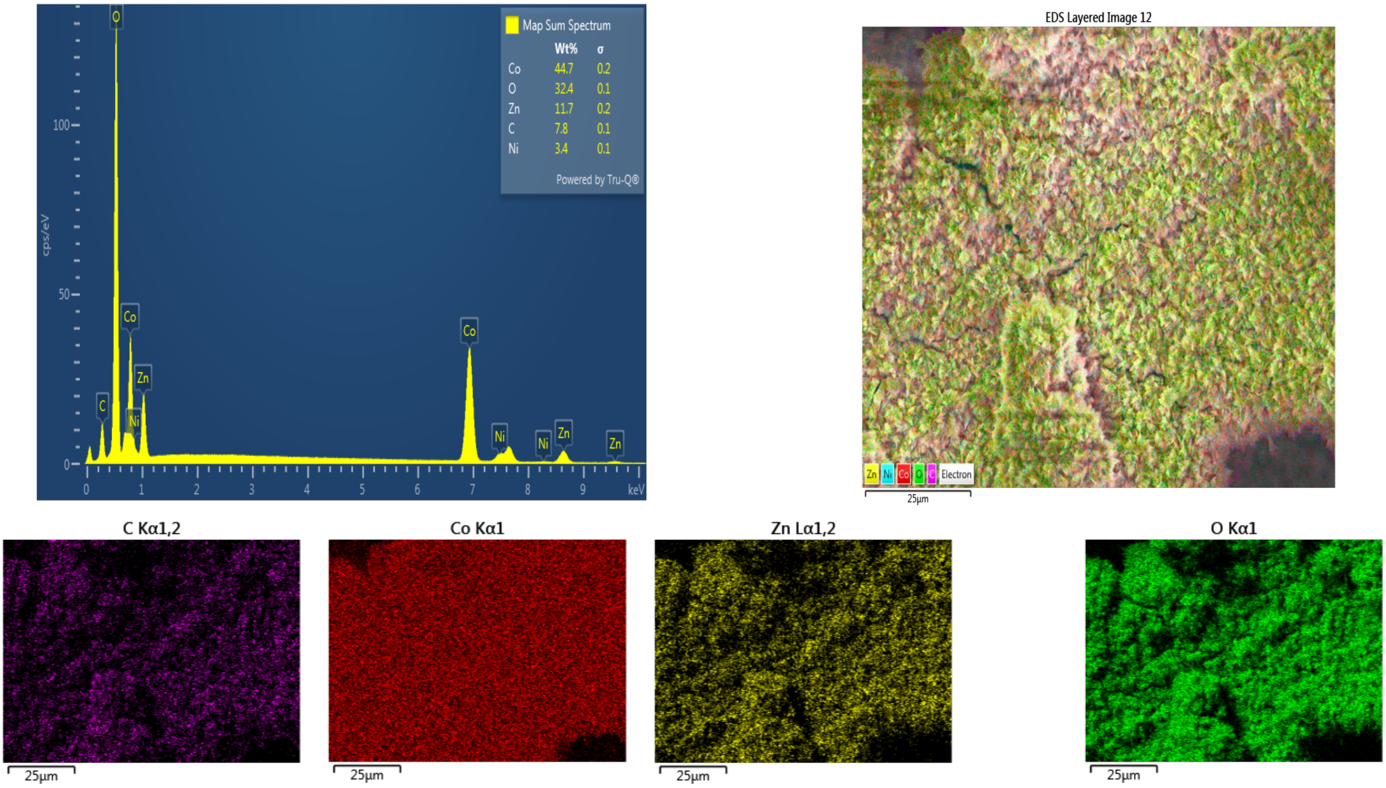

Fig. S3. EDS graph for sample #4.

nature portfolio

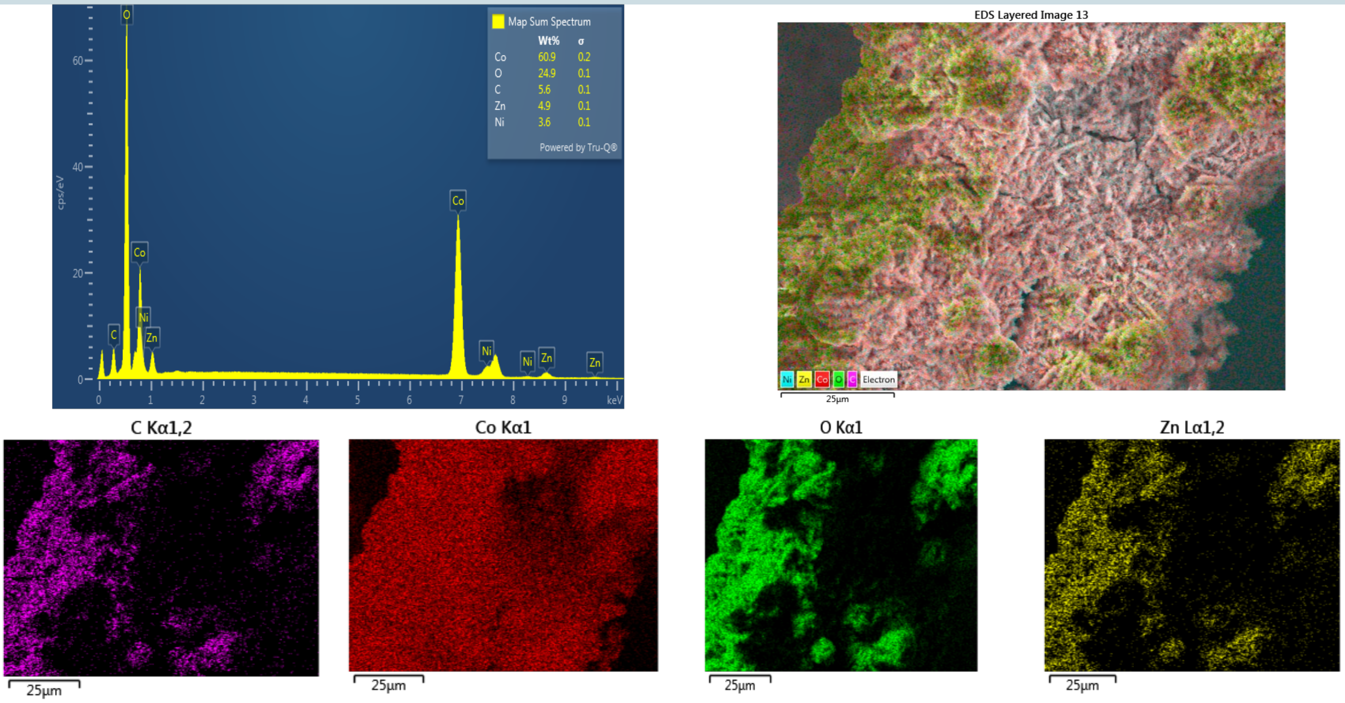

Fig. S4. EDS graph for sample #2.

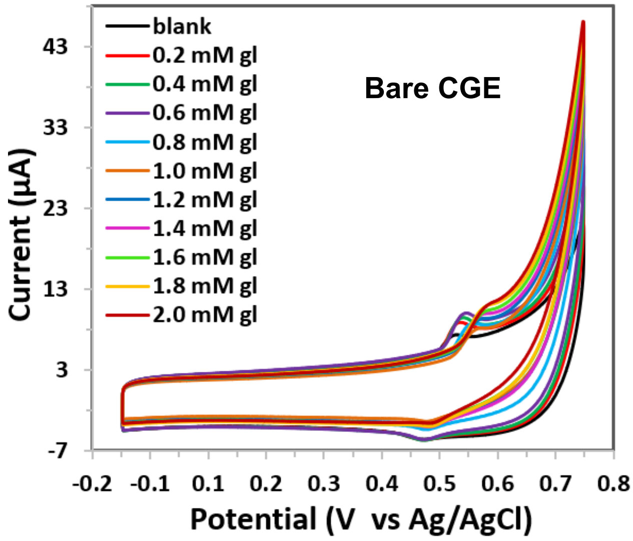

a

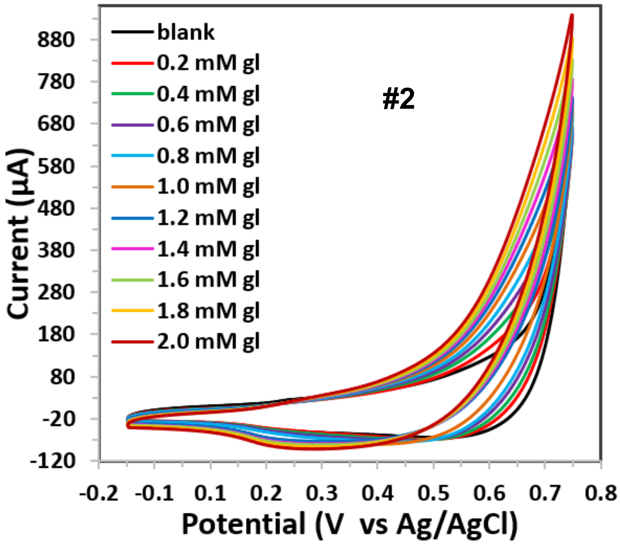

b

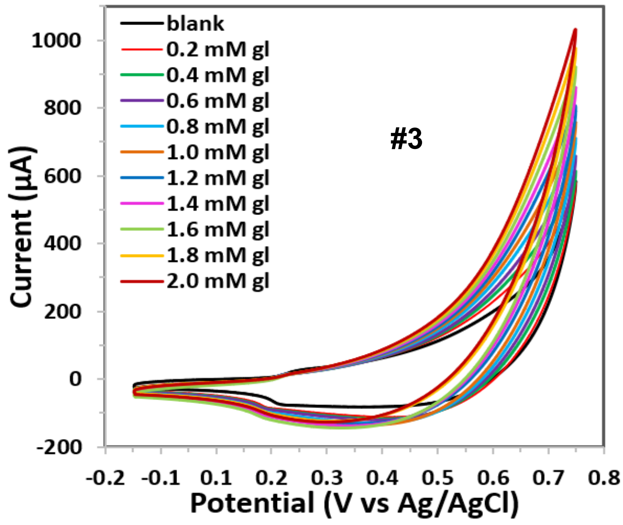

c

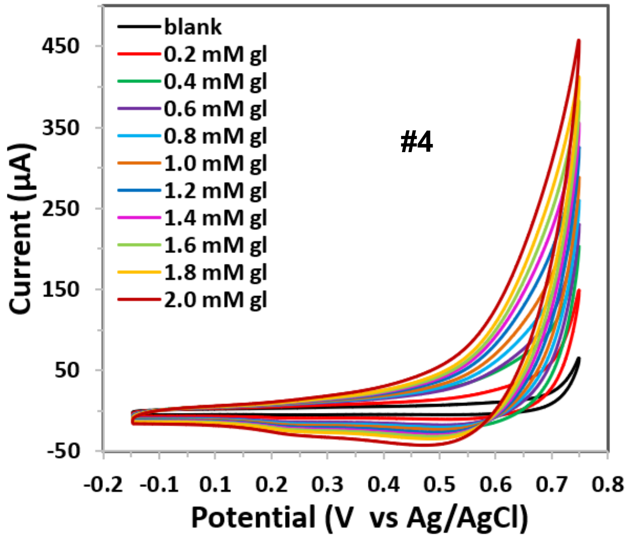

d

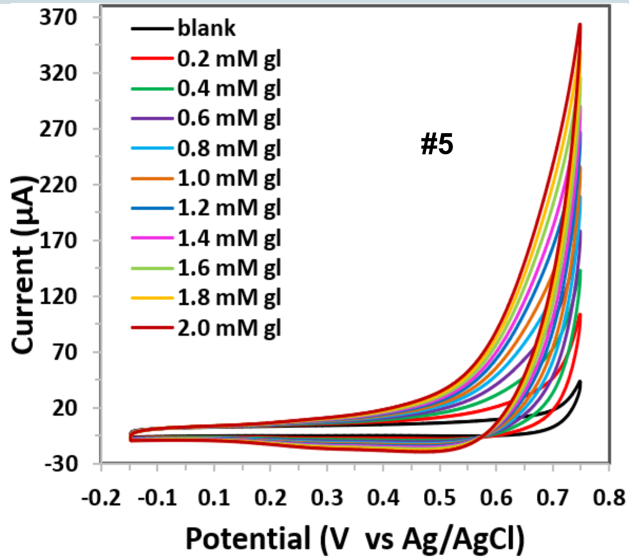
blank
0.2 mM gl
0.4 mM gl
0.6 mM gl
0.8 mM gl
1.0 mM gl
1.2 mM gl
1.4 mM gl
1.6 mM gl
1.8 mM gl
2.0 mM gl
#5
Current (μA)
370 320 270 220 170 120 70 20 -30
-0.2 -0.1 0.1 0.2 0.3 0.4 0.5 0.6 0.7 0.8
Potential (V vs Ag/AgCl)

e

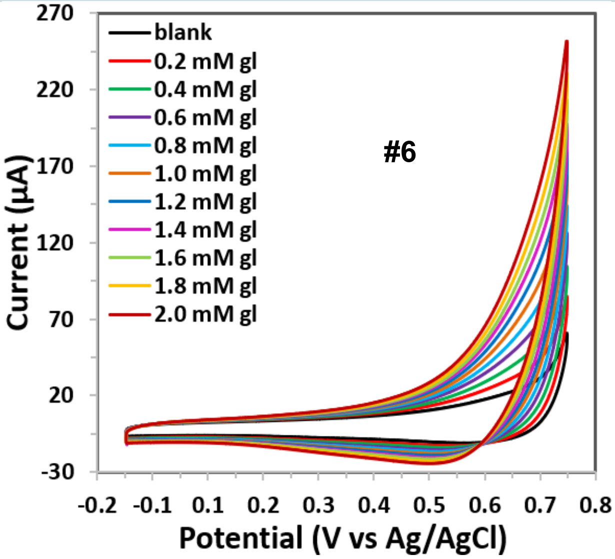
blank
0.2 mM gl
0.4 mM gl
0.6 mM gl
0.8 mM gl
1.0 mM gl
1.2 mM gl
1.4 mM gl
1.6 mM gl
1.8 mM gl
2.0 mM gl
#6
Current (μA)
270 220 170 120 70 20 -30
-0.2 -0.1 0.1 0.2 0.3 0.4 0.5 0.6 0.7 0.8
Potential (V vs Ag/AgCl)

f

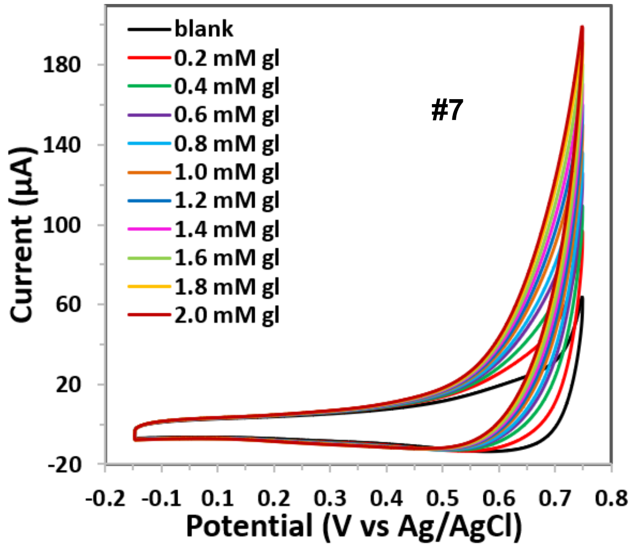
blank
0.2 mM gl
0.4 mM gl
0.6 mM gl
0.8 mM gl
1.0 mM gl
1.2 mM gl
1.4 mM gl
1.6 mM gl
1.8 mM gl
2.0 mM gl
#7
Current (μA)
180 140 100 60 20 -20
-0.2 -0.1 0.1 0.2 0.3 0.4 0.5 0.6 0.7 0.8
Potential (V vs Ag/AgCl)

g

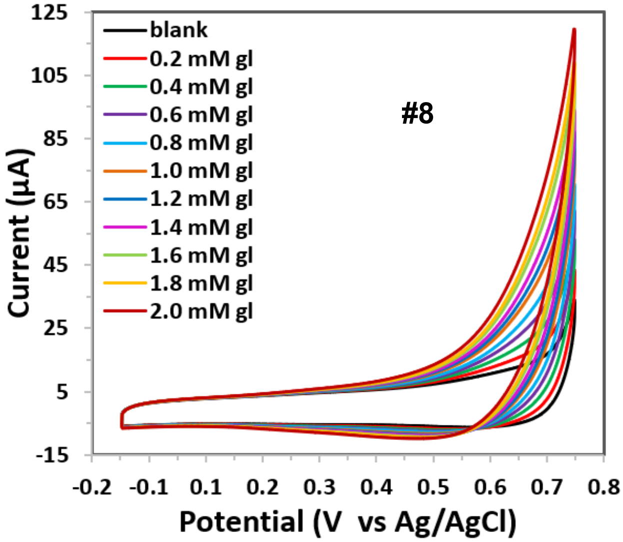
blank
0.2 mM gl
0.4 mM gl
0.6 mM gl
0.8 mM gl
1.0 mM gl
1.2 mM gl
1.4 mM gl
1.6 mM gl
1.8 mM gl
2.0 mM gl
#8
Current (μA)
125 105 85 65 45 25 5 -15
-0.2 -0.1 0.1 0.2 0.3 0.4 0.5 0.6 0.7 0.8
Potential (V vs Ag/AgCl)

h

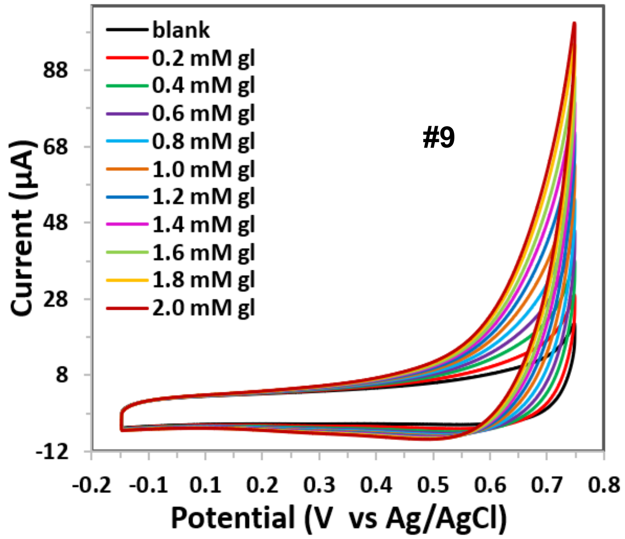
blank
0.2 mM gl
0.4 mM gl
0.6 mM gl
0.8 mM gl
1.0 mM gl
1.2 mM gl
1.4 mM gl
1.6 mM gl
1.8 mM gl
2.0 mM gl
#9
Current (μA)
88 68 48 28 8 -12
-0.2 -0.1 0.1 0.2 0.3 0.4 0.5 0.6 0.7 0.8
Potential (V vs Ag/AgCl)

i

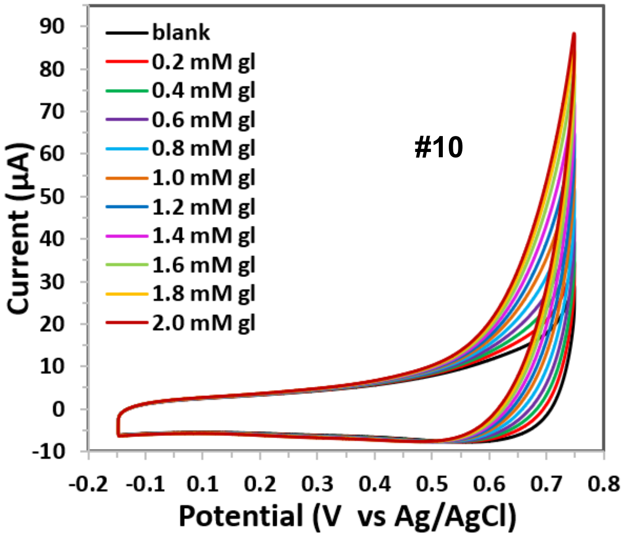
blank
0.2 mM gl
0.4 mM gl
0.6 mM gl
0.8 mM gl
1.0 mM gl
1.2 mM gl
1.4 mM gl
1.6 mM gl
1.8 mM gl
2.0 mM gl
#10
Current (μA)
90 80 70 60 50 40 30 20 10 0 -10
-0.2 -0.1 0.1 0.2 0.3 0.4 0.5 0.6 0.7 0.8
Potential (V vs Ag/AgCl)

j

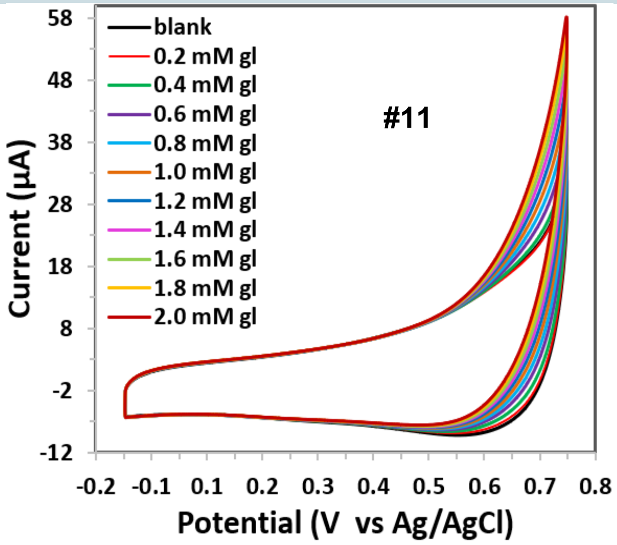

k

Fig. S5. Cyclic voltammetry of bare CGE and Zn dopped samples: a – bare CGE, b – 0.01, sample #2, c – 0.02, sample # 3, d – 0.05, sample #4, e – 0.09, sample #5, f – 0.14, sample #6, g – 0.2, sample #7, h – 0.33, sample # 8, i – 0.5, sample # 9, j – 0.67, sample #10, k – 0.9, sample # 11.

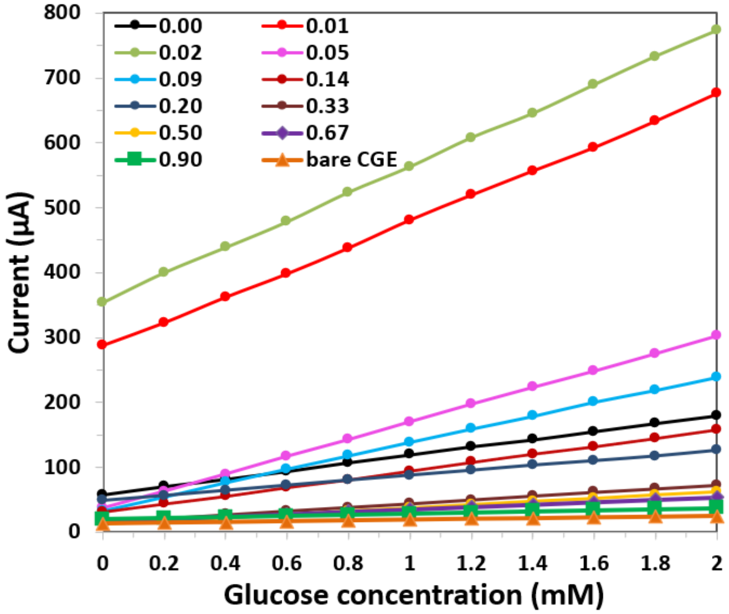

Fig. S6. Current increase at 0.6 V potential vs. Ag/AgCl as a function of glucose concentration for synthesized samples and for bare CGE electrode.

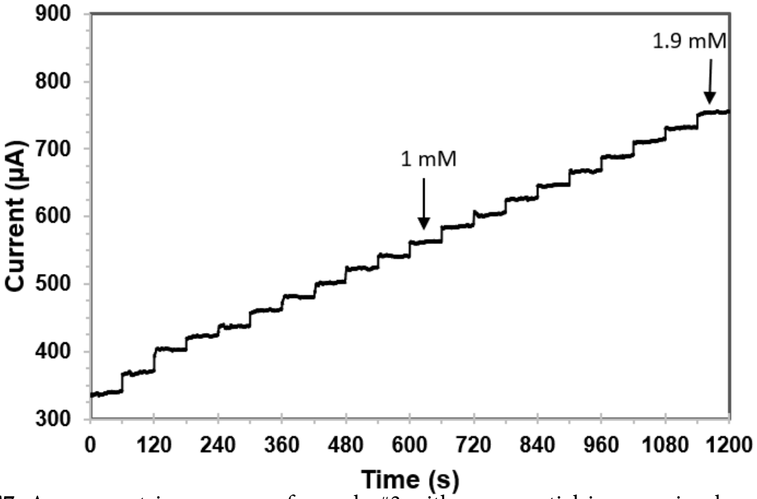

Fig. S7. Amperometric response of sample #3 with a sequential increase in glucose concentration by 0.1 mM increments.
